# Contrasting Effects of Fasting on Liver-Adipose Axis in Alcohol-Associated and Non-alcoholic Fatty Liver

**DOI:** 10.3389/fphys.2021.625352

**Published:** 2021-03-03

**Authors:** Karuna Rasineni, Clayton W. Jordan, Paul G. Thomes, Jacy L. Kubik, Elizabeth M. Staab, Sarah A. Sweeney, Geoffrey A. Talmon, Terrence M. Donohue, Mark A. McNiven, Kusum K. Kharbanda, Carol A. Casey

**Affiliations:** ^1^Department of Internal Medicine, University of Nebraska Medical Center, Omaha, NE, United States; ^2^Research Service, Veterans Affairs Nebraska-Western Iowa Health Care System, Omaha, NE, United States; ^3^Department of Pathology and Microbiology, University of Nebraska Medical Center, Omaha, NE, United States; ^4^Department of Biochemistry and Molecular Biology, University of Nebraska Medical Center, Omaha, NE, United States; ^5^Department of Biochemistry and Molecular Biology and the Center for Digestive Diseases, Mayo Clinic, Rochester, MN, United States

**Keywords:** alcohol-associated fatty liver disease, non-alcoholic fatty liver disease, starvation, hepatic lipid metabolism, adipose lipolysis, liver-adipose crosstalk

## Abstract

**Background:** Fatty liver, a major health problem worldwide, is the earliest pathological change in the progression of alcohol-associated (AFL) and non-alcoholic fatty liver disease (NAFL). Though the causes of AFL and NAFL differ, both share similar histological and some common pathophysiological characteristics. In this study, we sought to examine mechanisms responsible for lipid dynamics in liver and adipose tissue in the setting of AFL and NAFL in response to 48 h of fasting.

**Methods:** Male rats were fed Lieber-DeCarli liquid control or alcohol-containing diet (AFL model), chow or high-fat pellet diet (NAFL model). After 6–8 weeks of feeding, half of the rats from each group were fasted for 48 h while the other half remained on their respective diets. Following sacrifice, blood, adipose, and the liver were collected for analysis.

**Results:** Though rats fed AFL and NAFL diets both showed fatty liver, the physiological mechanisms involved in the development of each was different. Here, we show that increased hepatic *de novo* fatty acid synthesis, increased uptake of adipose-derived free fatty acids, and impaired triglyceride breakdown contribute to the development of AFL. In the case of NAFL, however, increased dietary fatty acid uptake is the major contributor to hepatic steatosis. Likewise, the response to starvation in the two fatty liver disease models also varied. While there was a decrease in hepatic steatosis after fasting in ethanol-fed rats, the control, chow and high-fat diet-fed rats showed higher levels of hepatic steatosis than pair-fed counterparts. This diverse response was a result of increased adipose lipolysis in all experimental groups except fasted ethanol-fed rats.

**Conclusion:** Even though AFL and NAFL are nearly histologically indistinguishable, the physiological mechanisms that cause hepatic fat accumulation are different as are their responses to starvation.

## Introduction

Fatty liver is a major health problem both in the United States and around the globe. It is the earliest and most common response to excessive ethanol consumption or over-consumption of a high-fat/high-sugar diet (Singal and Anand, 2013; Li et al., 2019; Wong et al., 2019; Mitra et al., 2020). It is characterized by the accumulation of fats, chiefly as triglycerides (TG), in the liver (Kharbanda et al., 2007a, 2012; Rasineni et al., 2016). Upon excessive lipid accumulation, the liver is susceptible to inflammatory mediators and toxic agents which cause progressive liver injury (Lieber, 2004; Gao et al., 2019). About 100 million individuals in the United States are estimated to have non-alcoholic fatty liver disease as a result of consumption of a high-fat/high-sugar diet, known as a Western diet (Cotter and Rinella, 2020; Cotter et al., 2020). It is estimated that 90–100% of alcohol consumers develop fatty liver (Singal et al., 2013; Tapper and Parikh, 2018). Fatty liver development after heavy alcohol consumption (AFL, alcohol-associated fatty liver) or that after high caloric-intake (NAFL, non-alcoholic fatty liver) have similar phenotypes, showing excessive hepatocellular lipid accumulation in the form of larger lipid droplets (Rasineni et al., 2016).

In the body, liver and adipose tissue play prominent roles in maintaining energy homeostasis in both fed and fasting states. Specifically, hepatocytes play an important role in glucose homeostasis by storing or producing glucose in response to a fed or fasted state (Klover and Mooney, 2004). Adipose tissue serves as a storage site for fat derived from excess food consumption. Fat is utilized to fulfill subsequent metabolic requirements to other tissues, including the liver. During times of little or no food consumption, adipose tissue releases non-esterified fatty acids (NEFA) into circulation (Tang et al., 2017). In the liver, NEFA can be oxidized by mitochondria or re-esterified to form TG within the endoplasmic reticulum to be stored in lipid droplets or secreted/exported into the blood as a component of very-low-density lipoprotein (VLDL) (Kawano and Cohen, 2013). Unlike adipose tissue, the liver is not intended as a storage site for excess lipids. However, ethanol misuse or the over-consumption of a high-fat/high-sugar diet increases hepatocyte TG accumulation, which leads to the development of fatty liver disease. Many mechanisms have been reported to play a role in the development of AFL, including increased flow of fatty acids to the liver from enhanced adipose tissue lipolysis (Wei et al., 2013), impaired fat transport out of the liver via reduced VLDL secretion (Kharbanda et al., 2009), increased lipogenesis and decreased fatty acid oxidation (Rasineni and Casey, 2012; Osna et al., 2017; You and Arteel, 2019). Similarly, several mechanisms have been proposed to contribute to the development of high-fat/high-sugar diet induced non-alcoholic hepatic steatosis, including carbohydrate and fatty acid substrate overload and/or impairment of fatty acid disposal pathways (Chao et al., 2019). Although the causes of AFL and NAFL are different, both share some common pathophysiological mechanism(s) for fat accumulation and are histologically similar (Rasineni et al., 2016).

Extensive studies conducted in our laboratory as well as others have shown that multiple pathways in adipose-liver crosstalk contribute to the development and progression of liver disease in both AFL and NAFL (Lomonaco et al., 2012; Rasineni et al., 2019b). It is also known that nutrient deprivation or fasting activates many tissue-specific metabolic pathways to provide energy to the body by coordination of organ-organ interactions. It is mainly liver and adipose tissue that act as a metabolic buffering system for adipose-released NEFA and its utilization by the liver to produce glucose for survival (Klover and Mooney, 2004; Tang et al., 2017). But how this liver-adipose axis is affected by fasting against a backdrop of hepatic fat accumulation remains unknown. Here, we sought to examine mechanisms involved in both AFL and NAFL to reveal similarities and differences between shared physiological pathways. Specifically, our study was aimed at deciphering the mechanism(s) responsible for lipid dynamics in liver and adipose tissue in the settings of AFL and NAFL and the response of each to a 48 h fast.

## Materials and Methods

### Reagents

Antibodies and reagents were purchased from the following companies: Ethanol was purchased from Pharmaco-AAPER (Brookfield, CT, United States). IRDye infrared secondary antibodies (Abs) and blocking buffer were from LI-COR Biosciences (Lincoln, NE, United States). Antibodies for adipose triacylglycerol lipase (ATGL), hormone sensitive lipase (HSL), and phosphorylated HSL (pHSL) were purchased from Cell Signaling (Danvers, MA, United States), phosphorylated pATGL was obtained from Abcam (Cambridge, MA, United States), and perilipin 2 (PLIN2) antibody was from Fitzgerald (Acton, MA, United States). All other chemicals were obtained from Sigma Chemical Co. (St. Louis, MO, United States) unless stated otherwise.

### Animal Maintenance and Tissue Collection

Male Wistar rats (175–200 g) were purchased from Charles River Laboratories (Portage, MI, United States). For the AFL model, rats were weight-matched and pair-fed Lieber-DeCarli ethanol liquid diet (Dyets Inc., Bethlehem, PA, United States; Cat# 710260; 18% of total energy as protein, 35% as fat, 11% as carbohydrates, and 36% as ethanol) or control diet (Cat# 710027; ethanol replaced isocalorically with maltodextrin) for 6–8 weeks as described previously (Rasineni et al., 2016). Rats were housed singly for the ethanol feeding experiments, as it is necessary to pair their feeding with control rats for proper experimental protocol. In addition, the rats in the AFL group were housed in wire-bottom cages so that the rats don’t eat their feces, a necessary component to control nutritional equivalence. This type of single caging has been utilized at the Omaha VAMC for alcohol feeding protocols and does not appear to add significant stress to the animals. For the NAFL model, rats were fed *ad libitum* either high-fat pellet diet (Research Diets #D08060104; 60% of calories derived from fat, 20% from carbohydrates, and 20% from protein) or maintained on standard chow pellet diet (Research Diets #D12450K; 10% of total calories from fat, 20% from protein, and 70% from carbohydrates as mainly corn starch, maltodextrin). NAFL and chow groups were allowed *ad libitum* access to their pellet diets and water (Rasineni et al., 2016). Rats in the NAFL model groups were housed in standard cages with bedding and a PVC pipe for enrichment. Forty-eight hours prior to termination of feeding, half of the rats in each of the four groups had their liquid or pellet diets removed. These rats were considered to be in fasting condition at the end of the 48 h time period (Cuervo et al., 1995; Schneider et al., 2014; Kaushik and Cuervo, 2015). These rats had *ad libitum* access to water during the fasting period. Note that chronic ethanol-fed rats did not exhibit any sudden alcohol withdrawal effects such as delirium tremens, hyperreactivity, psychological discomfort or death following the removal of alcohol diet during fasting. At the termination of the experimental period, all rats were anesthetized with isoflurane and blood was collected from the vena cava. Livers and epididymal adipose tissue were collected, rinsed in TE buffer (10 mM Tris–HCL, 1 mM EDTA) and weighed. Tissue sections were placed in 10% formalin for sectioning and remaining tissue was frozen in liquid nitrogen and stored at −80°C until further use. All animals received humane care in accordance with the guidelines established by the American Association for the Accreditation of Laboratory Animal Care. All protocols were approved by the Institutional Animal Care and Use Committee at the VA NWIHCS Research Service.

### Histology by H&E Staining

Paraffin-embedded liver and epididymal white adipose tissue was deparaffinized and stained with hematoxylin followed by counter-staining with eosin.

### Serum Analysis

Serum levels of alanine aminotransferase (ALT), alkaline phosphatase (ALP), and cholesterol were measured by a Vetscan chemistry analyzer (Abaxis, Union City, CA, United States) using a Mammalian Liver Profile reagent rotor (#89126-004). Serum non-esterified fatty acids (NEFA) were measured in an enzymatic colorimetric assay using a HR Series NEFA-HR2 kit (Wako Chemicals, Richmond, VA, United States). Serum triglycerides (TG) were quantified using a colorimetric diagnostic kit (#TR22421) from Thermo Fisher Scientific (Middletown, VA, United States).

### Hepatic TG

Measurement of liver TG were performed by lipid extraction of frozen liver pieces by the Folch procedure (Folch et al., 1957). Aliquots of the lipid extract were saponified to quantify the triglyceride mass using the triglyceride diagnostics kit (#TR22421) from Thermo Fisher Scientific (Middletown, VA, United States). Triglyceride levels were normalized to grams of liver tissue.

### Immunohistochemistry

Immunohistochemical staining for PLIN2, a lipid droplet protein, was conducted as described previously (Rasineni et al., 2014). Paraffin-embedded liver sections were deparaffinized, rehydrated and treated with 10 mM sodium citrate buffer (pH 6) for 20 min for antigen retrieval. Liver sections were incubated with a PLIN2 antibody (Fitzgerald#10R-A117ax) overnight followed by the appropriate Alexa Fluor secondary antibody for 1 h. Vectashield mounting medium with DAPI was used for mounting sections. Images were visualized and captured using a Keyence BZ-X series fluorescence microscope.

### Western Blot Analysis

Liver homogenates were prepared in TE buffer containing protease inhibitor cocktail (#P2714, Sigma, St. Louis, MO, United States). Liver samples were subjected to SDS-PAGE, followed by blotting of proteins to nitrocellulose membranes. The proteins were detected with specific primary antibodies and fluorescently labeled secondary antibodies. The proteins were visualized and quantified using the Odyssey Infrared Imager and associated software (LI-COR Biosciences, Lincoln, NE, United States).

### Messenger RNA Quantification

RNA was isolated from liver and epididymal adipose tissue using the PureLink RNA Mini Kit (Invitrogen, Carlsbad, CA, United States) and reverse-transcribed using the High Capacity cDNA Transcription Kit (Applied Biosystems, Carlsbad, CA, United States). Messenger RNAs were measured using iTaq Universal SYBR Green Supermix (Bio-Rad, Hercules, CA, United States) and primers from Integrated DNA Technologies (Coralville, IA, United States). PPARγ: Sense 5′- GAGATCCTCCTGTTGACCCAG-3′; Antisense 5′-CC ACAGAGCTGATTCCGAAGT-3′, PPARα: Sense 5′-GTCCT CTGGTTGTCCCCTTG-3′; Antisense 5′- GTCAGTTCACAGG GAAGGCA-3′, FAS: Sense 5′- TCCCAGGTCTTGCCGTGC-3′; Antisense 5′-GCGGATGCCTAGGATGTGTGC-3′, FATP2: Sense 5′- AGTACATCG GTGAACTGCTTCGGT-3′; Antisense 5′-TGCCTTCAGTGGAAGCGTAGAACT-3′, CD36: Sense 5′-AACCCAGAGGAAGTGGCAAAG-3′; Antisense 5′-GACAG TGAAGGCTCAAAGATGG-3′. Samples were analyzed using the 7500 Real Time PCR System (Applied Biosystems, Carlsbad, CA, United States). We used rat-specific primers from Applied Biosystems for mRNA analysis of monocyte chemoattractant protein-1 (MCP-1/CCL2; catalog #Rn00580555) and interleukin 1 beta (IL-1β; catalog #Rn00580432). The ΔΔCt method was used to determine the fold change of each transcript using β-actin or 36B4 mRNA for normalization.

### Statistical Analysis

Results are expressed as mean values ± SEM. Comparison between fed and fasted animals in AFL and NAFL groups were analyzed using the Student’s *t*-test. *p*-values of <0.05 were considered significant. For statistical analyses, data from ethanol-fed rats and their fasted cohorts were compared with pair-fed control rats. In the NAFL group, HFD-fed rats and their fasted cohorts were compared with chow-fed rats.

## Results

### Liver and Adipose Weight in Fed and Fasted Rats

**FIGURE 1:**
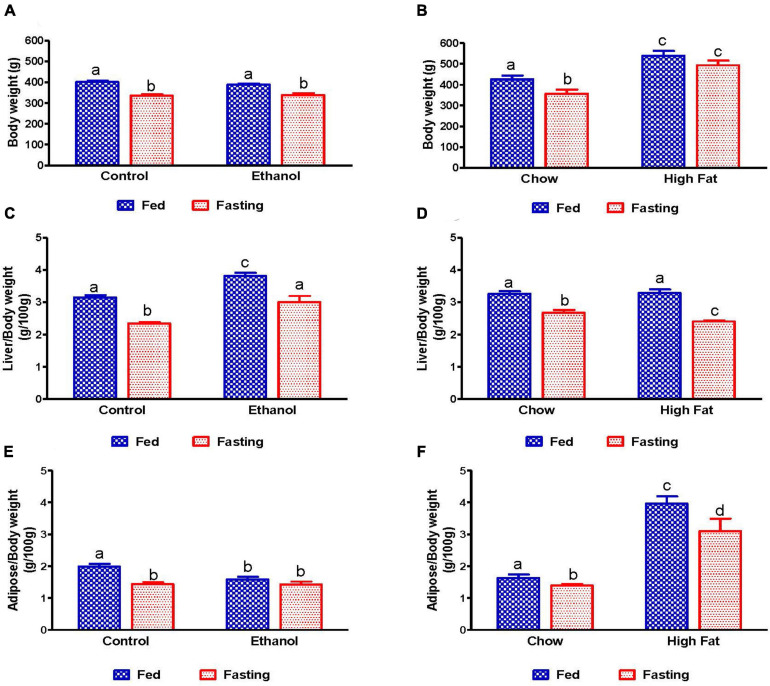
Body, liver, and adipose weight in fed and fasting AFL and NAFL experimental animals. Body weights of **(A)** Lieber-DeCarli pair-fed control and ethanol-fed rats (AFL) and their respective fasted counterparts, **(B)** chow or high-fat diet (HFD) fed rats (NAFL) and their respective fasted groups. Liver weights of **(C)** AFL group and **(D)** NAFL group. Adipose weights of **(E)** AFL group and **(F)** NAFL group. Values are means ± SEM (*n* = 8). Values not sharing a common letter in each group are statistically different, *p* < 0.05.

As shown in Figure 1A, with pair-feeding, we observed similar body weights in the ethanol-fed and pair-fed control rats. While we did not observe any difference in the average caloric intake per day in the NAFL model groups (chow-fed rats:100.6 ± 6.93 Kcal/day; HFD-fed rats:114.1 ± 10.94 Kcal/day; *n* = 8; data expressed as mean ± SEM), we did see significantly higher body weights in HFD-fed rats compared to chow-fed rats (Figure 1B). Forty-eight hours of fasting significantly reduced the body weights of rats in all experimental groups compared with their respective fed-counterparts, except for the HFD group. Furthermore, consistent with our previous study (Rasineni et al., 2019b), liver weights were significantly higher and adipose weights lower in ethanol-fed rats, resulting in an increased liver/body weight ratio (Figure 1C) and decreased adipose/body weight ratio (Figure 1E) as compared to their respective controls. Interestingly, in HFD-fed rats, both liver and body weights were increased, resulting in no change in the liver/body weight ratio when compared to the chow-fed rats (Figure 1D). However, we did observe significantly higher epididymal adipose weight and consequently higher epididymal/body weight ratios in HFD-fed rats compared with chow-fed rats (Figure 1F). As shown in Figures 1C,D, 48 h fasting decreased liver weight, resulting in a decline in liver/body weight ratio in all experimental groups. In addition, we observed a significant decrease in adipose weight with 48 h fasting in all experimental groups except ethanol-fed rats (Figures 1E,F).

### Activity of Hepatic Injury Marker Enzymes and Lipid Concentrations in Serum

**FIGURE 2:**
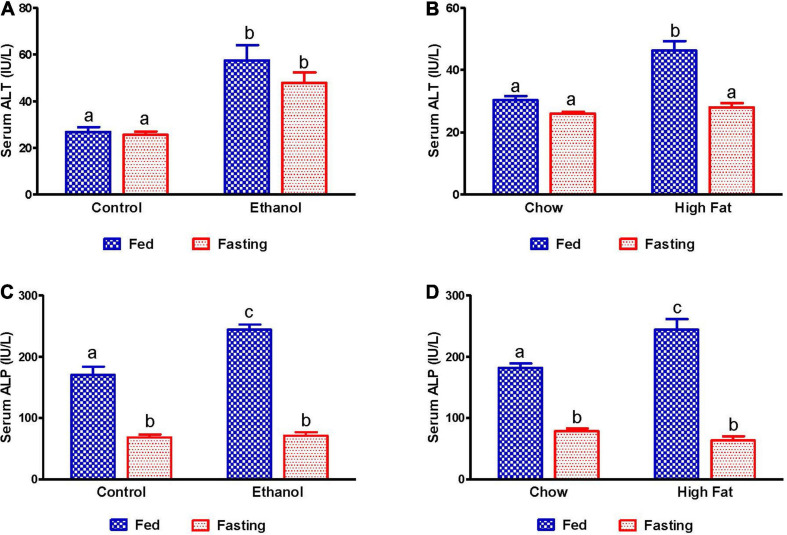
Liver injury markers in serum of AFL and NAFL experimental groups. Hepatic injury markers ALT in **(A)** Lieber-DeCarli pair-fed control and ethanol-fed rats (AFL) and their respective fasted counterparts, **(B)** chow or high-fat diet (HFD) fed rats (NAFL) and their respective fasted groups. ALP in **(C)** AFL group and **(D)** NAFL group. Values are means ± SEM (*n* = 8). Values not sharing a common letter in each group are statistically different, *p* < 0.05.

Since studies from our group and others have reported that the activity of serum hepatic injury markers increases during the development of both AFL and NAFL, we measured serum alanine aminotransferase (ALT) and alkaline phosphatase (ALP) to investigate the effect of 48 h fasting on hepatic injury. As expected, activity of serum ALT and ALP increased in both ethanol and HFD-fed rats compared with pair-fed control and chow-fed rats, respectively (Figure 2). Whereas 48 h fasting caused a significant ALT decrease in only the HFD-fed group (Figures 2A,B), serum ALP levels decreased in all experimental groups compared to their respective fed-counterparts (Figures 2C,D).

**FIGURE 3:**
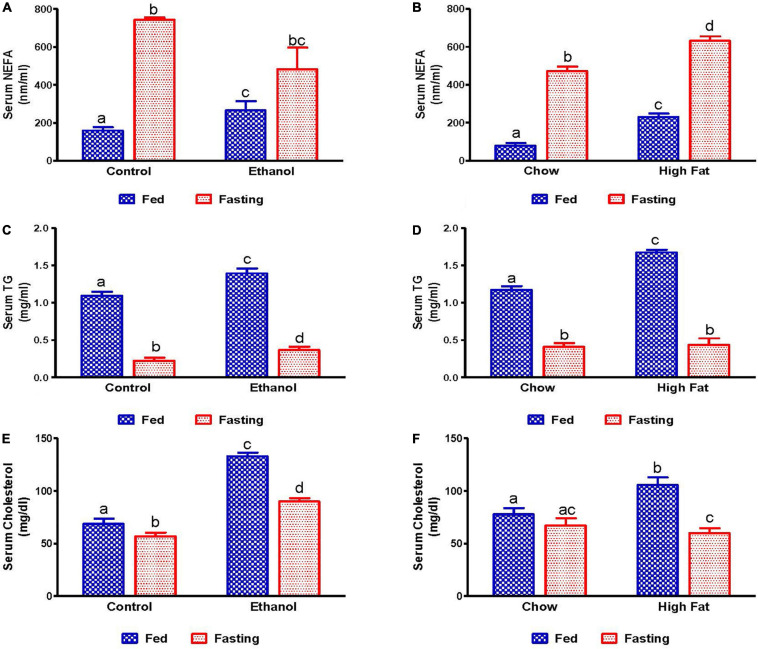
Serum lipid content in fed and fasting states of AFL and NAFL model animals. Serum NEFA in **(A)** Lieber-DeCarli pair-fed control and ethanol-fed rats (AFL) and their respective fasted counterparts, **(B)** chow or high-fat diet (HFD) fed rats (NAFL) and their respective fasted groups. Serum TG in **(C)** AFL group and **(D)** NAFL group. Serum cholesterol in **(E)** AFL group and **(F)** NAFL group. Values are means ± SEM (*n* = 8). Values not sharing a common letter in each group are statistically different, *p* < 0.05.

We also measured serum lipid content in all experimental groups. Serum NEFA, TG, and total cholesterol levels were higher in ethanol and HFD-fed rats compared with their respective controls (Figures 3A–F). As expected, 48 h fasting increased circulating NEFA levels in all experimental groups, except the ethanol group, relative to corresponding fed groups (Figures 3A,B). In contrast to serum NEFA levels, fasting significantly decreased serum TG and cholesterol levels in all experimental groups compared with fed groups (Figures 3C–F).

### Morphology of Liver Tissue and Hepatic TG Levels in Fed and Fasted AFL and NAFL Rats

**FIGURE 4:**
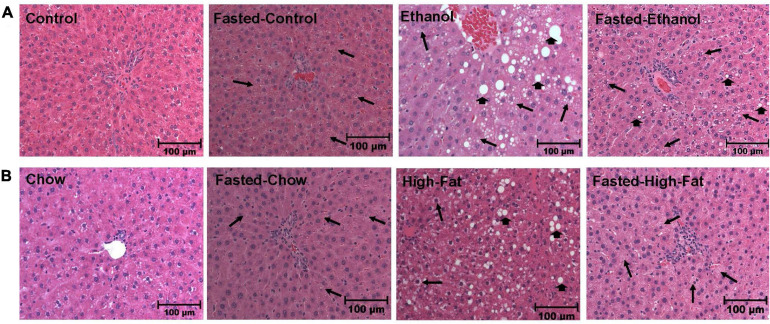
Morphology of liver tissue. Hematoxylin and eosin staining was performed on paraffin sections of fed and fasted ethanol- and high-fat diet (HFD)-fed rats and their respective controls. Images are representative of each group (*n* = 8). Magnification, 200X. **(A)** shows Lieber-DeCarli pair-fed control and ethanol-fed rats (AFL) and their respective fasted counterparts. **(B)** shows chow or high-fat diet (HFD)-fed rats (NAFL) and their respective fasted groups. Macrovesicular steatosis is denoted by arrowhead while arrow depict microvesicular steatosis.

**FIGURE 5:**
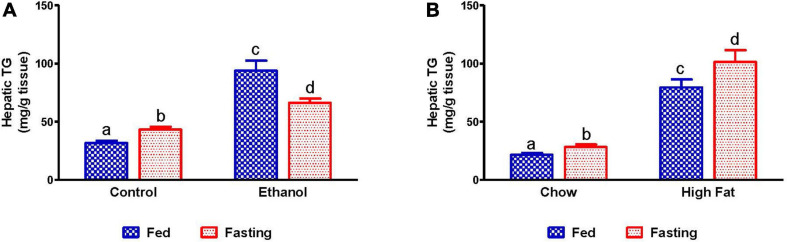
Quantification of lipid content in liver. Hepatic TG in **(A)** pair-fed control and ethanol Lieber-DeCarli liquid diet-fed rats (AFL) and their respective fasted counterparts, **(B)** chow or high-fat diet (HFD) fed rats (NAFL) and their respective fasted groups. Values are means ± SEM (*n* = 8). Values not sharing a common letter in each group are statistically different, *p* < 0.05.

**FIGURE 6:**
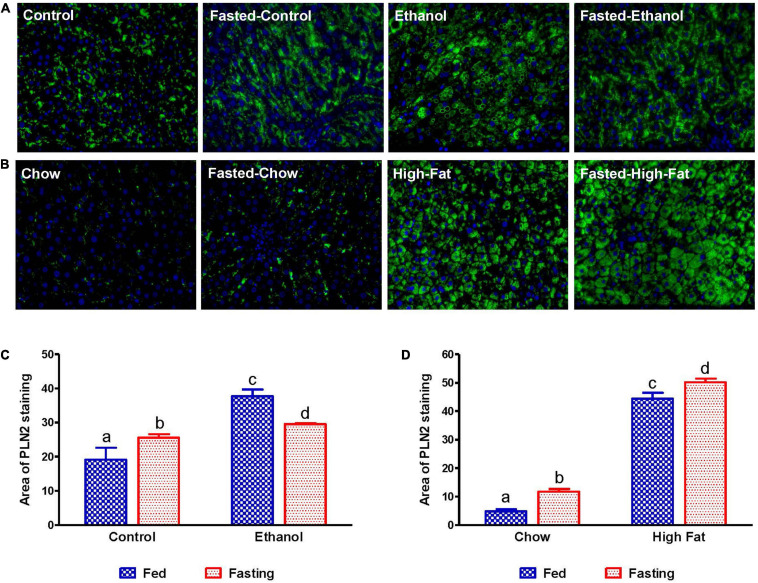
Immunohistochemical staining for lipid droplet-associated protein PLIN2. Liver sections from animals were stained with perilipin 2 (PLIN2) antibody. **(A)** Lieber-DeCarli pair-fed control and ethanol-fed rats (AFL) and their respective fasted counterparts. **(B)** chow or high-fat diet-fed rats (NAFL) and their respective fasted groups. Images are representative of each group of *n* = 8. Magnification, 400×. The histogram represents the area of PLIN 2 staining in **(C)** AFL group and **(D)** NAFL group. Values are means ± SEM (*n* = 8). Values not sharing a common letter in each group are statistically different, *p* < 0.05.

We collected livers from fed and fasted rats to examine fat content by both histological and biochemical assessments. The histopathological evaluations of the formalin-fixed H&E stained liver sections were consistent with previous reports in that both alcohol and HFD-fed rats showed higher hepatic fat accumulation with evidence of both micro and macrovesicular steatosis as compared with their respective controls (Figures 4A,B). Since we observed decreased liver/body weight ratios, we expected lower hepatic lipid accumulation in fasted rats compared with respective fed animals. However, all except ethanol-fed rats showed higher hepatic fat with predominantly microvesicular steatosis compared with respective fed groups after 48 h fasting (Figures 4A,B). Interestingly in ethanol-fed rats, 48 h fasting decreased hepatic lipid accumulation, showing mostly microvesicular steatosis. These histological findings were corroborated by quantification of hepatic TG levels (Figures 5A,B) showing, in most cases, that fasting increased TG levels in the liver. Further, we stained hepatic sections for lipid droplet associated protein, perilipin2 (PLIN2). Perilipins are one of the most abundant lipid droplet proteins (Sztalryd and Brasaemle, 2017). Among them, perilipin2 is ubiquitously expressed and exclusively associated with lipid droplets (LDs) as reported previously (Rasineni et al., 2014, 2016). As shown in Figures 6A–D, with quantitative analysis, we observed a marked increase in PLIN2 staining of ethanol and HFD-fed rat LDs. Apart from the ethanol-fed rats, all groups showed increased PLIN2 staining following 48 h fasting. The area of PLIN2 staining was concurrent with the histology (Figures 4A,B) and hepatic TG levels (Figures 5A,B).

### Hepatic Fatty Acid Synthesis, Break Down and β-Oxidation in Fed and Fasted States

**FIGURE 7:**
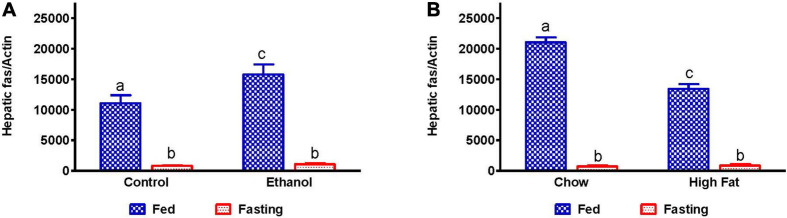
Hepatic fatty acid synthesis in AFL and NAFL animals during fed and fasting conditions. Hepatic expression of fatty acid synthase (FAS), an enzyme which regulates fatty acid synthesis. FAS in **(A)** Lieber-DeCarli pair-fed control and ethanol-fed rats (AFL) and their respective fasted counterparts, **(B)** chow or high-fat diet (HFD) fed rats (NAFL) and their respective fasted groups. Values are means ± SEM (*n* = 8). Values not sharing a common letter in each group are statistically different, *p* < 0.05.

We measured mRNA that encodes fatty acid synthase (FAS), which catalyzes fatty acid synthesis. Because we observed elevated TG in the livers of alcohol and HFD-fed rats, we expected higher levels of FAS mRNA. While ethanol-fed rats showed higher FAS mRNA expression than their pair-fed controls, HFD-fed rats showed lower FAS mRNA levels than respective controls, suggesting that lipogenic pathways are upregulated during the development of AFL (Figures 7A,B), but not NAFL. Fasting significantly decreased FAS mRNA in all experimental groups (Figures 7A,B).

**FIGURE 8:**
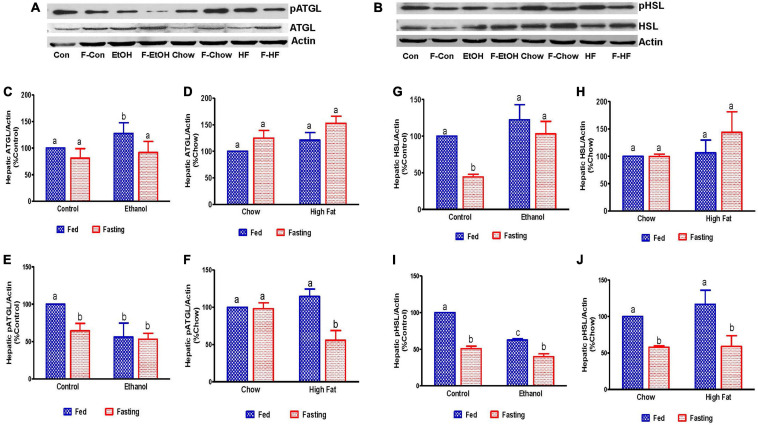
Estimation of hepatic enzymes involved in the regulation of hepatic triglyceride breakdown. Representative Western Blot from AFL and NAFL groups for **(A)** adipose triglyceride lipase (ATGL) and **(B)** hormone-sensitive lipase (HSL). **(C–F)** Panels of densitometric values of total and active (phosphorylated) content of ATGL in AFL and NAFL groups. **(G–J)** Panels of densitometric values of total and active HSL in AFL and NAFL groups. Values are means ± SEM (*n* = 6). Values not sharing a common letter in each group are statistically different, *p* < 0.05. Con, control; F-Con, fasted control; EtOH, ethanol; F-EtOH, fasted ethanol; Chow, chow fed; F-Chow, fasted chow; HF, high-fat-fed; F-HF, fasted high-fat-fed group.

**FIGURE 9:**
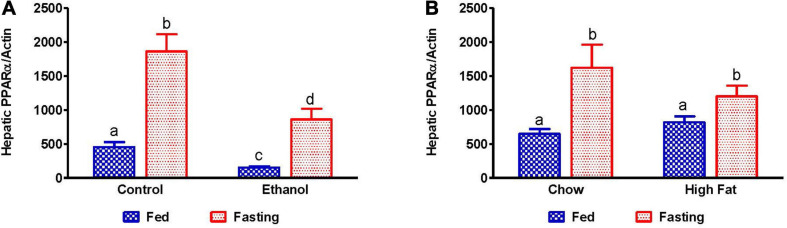
Expression of peroxisome proliferator-activated receptor α (PPARα). qPCR analysis of hepatic gene expression related to fatty acid oxidation, PPARα. **(A)** PPARα in Lieber-DeCarli pair-fed control and ethanol-fed rats (AFL) and their respective fasted counterparts, **(B)** chow or high-fat diet (HFD) fed rats (NAFL) and their respective fasted cohorts. Values are means ± SEM (*n* = 8). Values not sharing a common letter in each group are statistically different, *p* < 0.05.

Since fat accumulation represents either increased fat synthesis and/or lower fat catabolism, we measured the activities of two main lipases, adipose triacylglycerol lipase (ATGL) and hormone sensitive lipase (HSL), which catalyzes triglyceride breakdown. Western blot analysis (Figures 8A–J) revealed no significant change in either total ATGL (Figures 8C,D) or HSL (Figures 8G,H) protein content in the livers of ethanol or HFD-fed rats. However, we did observe a significant decrease in the active forms of ATGL (pATGL) and HSL (pHSL) in ethanol-fed rats compared with their pair-fed controls (Figures 8E,I). Notably, we observed no significant changes in either pATGL or pHSL in HFD-fed rats compared to chow-fed rats (Figures 8F,J). Fasting significantly decreased pATGL in rats fed the Lieber-DeCarli control diet and HFD, but not in the ethanol or chow-fed rats. However, active HSL was significantly decreased in all fasted animals compared to respective fed groups. Further, we measured hepatic peroxisome proliferator-activated receptor alpha, PPARα, a ligand-activated transcription factor involved in fatty acid oxidation. As expected, PPARα in ethanol-fed rats was lower than in pair-fed controls (Figure 9A). In HFD-fed rats, however, we observed no significant change in PPARα expression when compared to chow-fed rats (Figure 9B). Since fatty acids are a major source of energy during starvation, 48 h fasting increased PPARα expression in all experimental groups, as expected (Figures 9A,B).

### Hepatic Fatty Acid Uptake

**FIGURE 10:**
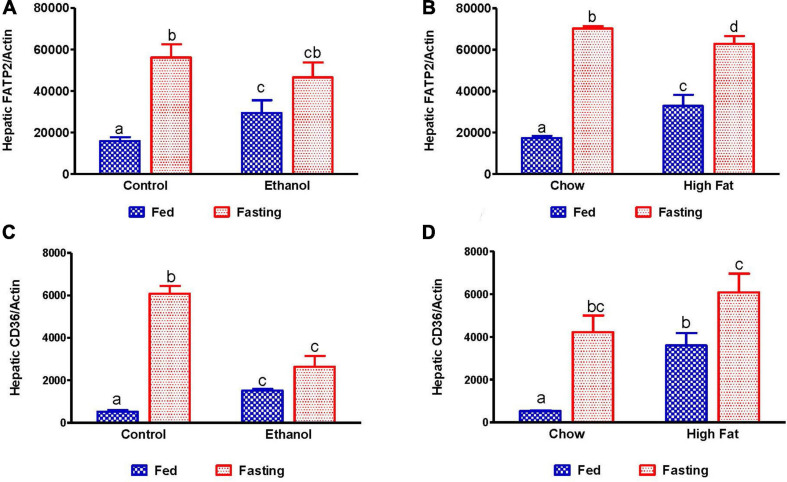
Quantitative analysis of hepatic fatty acid uptake. Hepatic gene expression related to fatty acid uptake. Fatty acid transport protein 2 (FATP2) in **(A)** Lieber-DeCarli pair-fed control and ethanol-fed rats (AFL) and their respective fasted counterparts, **(B)** in chow or high-fat diet (HFD) fed rats (NAFL) and their respective fasted cohorts. Fatty acid translocase CD36 in **(C)** AFL group and **(D)** NAFL group. Values are means ± SEM (*n* = 8). Values not sharing a common letter in each group are statistically different, *p* < 0.05.

Several studies have reported that adipose-derived free fatty acids are taken up by the liver and esterified to form TG, which can be utilized either for energy production, gluconeogenesis or stored in lipid droplets during fatty liver development. Several proteins are responsible for hepatic fatty acid uptake/transport, including fatty acid transport protein 2 (FATP2, a member of the FATP family of fatty acid uptake mediators) and cluster of differentiation 36 (CD36, a long-chain fatty acid-translocase). As expected, we observed increased expression of hepatic FATP2 and CD36 in ethanol and HFD-fed rats compared with pair-fed control and chow-fed rats, respectively (Figures 10A–D). Except for ethanol-fed rats, 48 h fasting increased circulating NEFA in parallel with a significant elevation of CD36 expression compared with respective fed-counterparts, indicating that fasting increases both adipose lipolysis and hepatic uptake of circulating fatty acids (Figures 10A–D). Similar increases in FATP2 were observed in all groups except for ethanol-fed animals following 48 h fasting.

### Hepatic Inflammation

**FIGURE 11:**
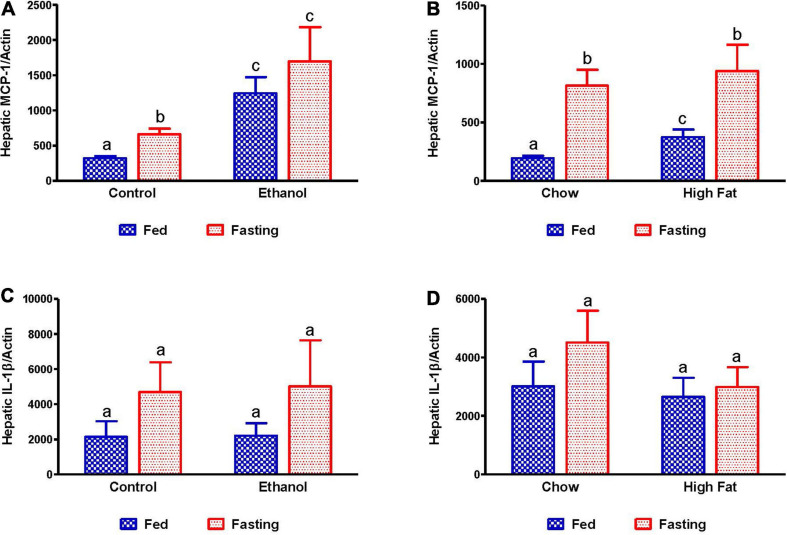
Expression of hepatic inflammatory markers. qPCR analysis of hepatic gene expression related to inflammation and steatosis. Monocyte chemoattractant protein-1 (MCP-1/CCL2) in **(A)** Lieber-DeCarli pair-fed control and ethanol-fed rats (AFL) and their respective fasted counterparts, **(B)** chow or high-fat diet-fed rats (NAFL) and their respective fasted cohorts. Interleukin 1 beta (IL-1β) in **(C)** AFL group and **(D)** NAFL group. Values are means ± SEM (*n* = 8). Values not sharing a common letter in each group are statistically different, *p* < 0.05.

Since it has been reported that fatty liver can promote inflammation (Mandrekar et al., 2011), which is a key player in progression of both alcoholic and non-alcoholic liver diseases (Gao and Tsukamoto, 2016), we examined the expression of inflammatory markers, monocyte chemoattractant protein 1 (MCP-1), and interleukin 1 beta (IL-1β). As shown in Figure 11, we observed increased MCP-1 in alcohol and HFD-fed rats compared to respective controls. Fasting for 48 h increased MCP1 in all experimental groups relative to respective controls, except for the ethanol-fed group (Figures 11A,B). No significant elevation of IL-1β was observed in either the ethanol or HFD-fed rats compared to respective controls nor did 48 h fasting induce IL-1β expression in any experimental groups.

### Fatty Acid Storage and Fatty Acid Breakdown in Adipose Tissue

**FIGURE 12:**
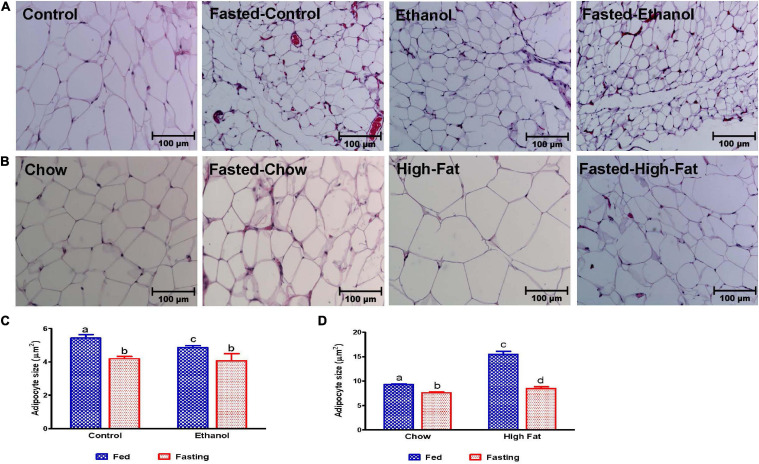
Morphology of epididymal adipose tissue. Hematoxylin and Eosin staining was performed on paraffin sections. Images are representative of each group of *n* = 8. Magnification 200X. **(A)** Lieber-DeCarli pair-fed control and ethanol-fed rats (AFL) and their respective fasted counterparts. **(B)** chow or high-fat diet (HFD) fed rats (NAFL) and their respective fasted groups. Average adipocyte size in **(C)** AFL group and **(D)** NAFL group. ImageJ software (National Institutes of Health, Bethesda, MD, United States) was used to measure average adipocyte size. Values are means ± SEM of four randomly selected fields from each section, and data pooled from sections obtained from four animals from each experimental group. Values not sharing a common letter in each group are statistically different, *p* < 0.05.

**FIGURE 13:**
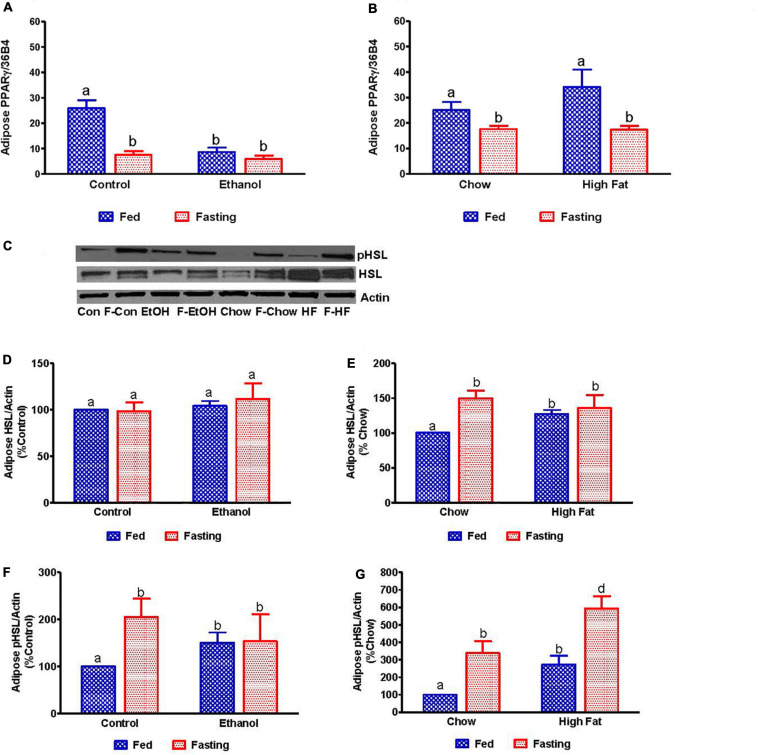
Quantitative analysis of the adipose fatty acid breakdown. qPCR analysis of peroxisome proliferator-activated receptor γ (PPARγ) in **(A)** Lieber-DeCarli pair-fed control and ethanol-fed rats (AFL) and their respective fasted counterparts, **(B)** in chow or high-fat diet (HFD) fed rats (NAFL) and their respective fasted cohorts. For quantitative analysis of adipose lipid breakdown, we estimated total and phosphorylated hormone-sensitive lipase (HSL). **(C)** Representative Western Blot. **(D,E)** Panel of densitometric values of total HSL in AFL and NAFL groups. **(F,G)** Panel of densitometric values of active (phosphorylated) HSL in AFL and NAFL groups. Values are means ± SEM (*n* = 6). Values not sharing a common letter in each group are statistically different, *p* < 0.05. Con, control-fed; F-Con, fasted control; EtOH, ethanol-fed; F-EtOH, fasted ethanol; Chow, chow-fed; F-Chow, fasted chow; HFD, high-fat diet-fed; F-HFD, fasted high-fat diet group.

We collected the epididymal white adipose tissue (eWAT) from fed and fasted animals to examine adipocyte size, which is representative of fat content. The histological evaluations of the formalin-fixed H&E stained eWAT revealed a decrease in the adipocyte size of ethanol-fed rats compared with controls, which is consistent with previous reports (Zhong et al., 2012). HFD-fed rats showed larger adipocyte size compared to chow-fed controls as previously reported (Li et al., 2016; Figures 12A–D). All fasted rats showed decreased adipocyte size compared with respective fed groups (Figures 12C,D). Further, we measured PPARγ (a transcription factor that induces adipose differentiation and increases fat storage capacity) and HSL (an enzyme that catalyzes triglyceride breakdown). As reported previously, in ethanol-fed rats we observed lower adipose PPARγ (Figure 13A) and higher HSL levels compared with pair-fed controls (Figures 13C,F). In HFD-fed rats, parallel to increased eWAT weight, we observed higher adipose PPARγ (Figure 13B) and HSL expression compared with chow-fed rats (Figures 13E,G). PPARγ expression declined in all groups after 48 h fasting. Whereas fasting significantly increased adipose HSL activity in rats fed the Lieber-DeCarli control, chow or high-fat diet, this same increase was not observed in adipose tissue of ethanol-fed rats (Figures 13F,G).

## Discussion

Fatty liver arising from either excessive alcohol consumption or over-consumption of a high-fat/high-sugar diet have similar phenotypes of hepatic lipid accumulation In our previous studies, we reported that despite showing similar phenotypic characteristics of accumulation of hepatic lipids and increased oxidative stress in livers of rats with AFL or NAFL, though there are mechanistic differences including impairment in membrane trafficking, which is observed only in AFL development (Rasineni et al., 2016). Here, we sought to evaluate the effect of fasting on lipid dynamics and metabolism by focusing on the adipose-liver axis in both models of fatty liver disease.

As expected, HFD-fed rats exhibited increased body weight and adipose/body weight ratios but had no change in liver/body weight ratio compared to the chow-fed group. In the case of HFD-fed rats, excessive nutrient/caloric intake results in additional energy being stored in adipose tissue in the form of lipids. This results in an increased adipose, body weight, and adipose/body weight ratio compared with the chow group. Further, when adipose tissue exceeds its ability to store lipids, NEFA flow to other organs increases, especially to the liver, leading to increased fat deposition and liver weight (Langin, 2011). Since there was an increase in both the liver and body weight of rats fed the HFD, the liver/body weight ratio in these rats did not differ from the chow-fed controls. However, despite weighing ∼100 g less than HFD-fed rats, ethanol-fed rats showed similar body weights to pair-fed controls, but markedly decreased adipose/body weight ratios as adipose lipolysis increased. As a result, the ethanol-fed rats exhibited increased liver/body weight ratios partly due to protein (Kharbanda et al., 2007b) and fat accumulation in the liver compared with respective controls (Rasineni et al., 2014). The latter occurred as a result of the combined effect of alcohol-induced *de novo* lipogenesis and adipose-derived fatty acid uptake and esterification as well as decreased fatty acid oxidation and VLDL secretion (Kharbanda et al., 2009; Rasineni et al., 2019a). Fasting for 48 h caused a significant decrease in body weights of all experimental animals except HFD-fed rats, which is consistent with reports in the literature (Li and Wassner, 1984). It has been shown that the extent of weight gain and body fat accumulation in older rats can regulate body weight loss following food deprivation. Hence, percentage of body weight loss is greater in younger rats than in older rats after 24 h of starvation (Li and Wassner, 1984). Our results were more consistent with liver weights which decreased in all experimental groups after the 48 h fast. This fasting-induced decrease in liver weights is likely due to breakdown and utilization of glycogen, which is an immediate and effective energy source to maintain blood glucose during the early stages of fasting (Ohama et al., 1994; Klover and Mooney, 2004), combined with the considerable amount of glycogen-associated water loss (Sharma et al., 1985). While glycogen is utilized during early periods of starvation, 48 h fasting results in the breakdown of adipose-stored TG for energy. Indeed, adipose/body weight ratios significantly decreased following fasting, indicating increased adipose lipolysis in all experimental groups except ethanol-fed rats. This was also indicated by increased circulating NEFA levels in all fasted groups except ethanol-fed rats. This discrepancy with the ethanol-fed rats is likely due to already accelerated lipolysis and perhaps the inability of adipose tissue to respond adequately to the fasting stimulus and release NEFA to the extent observed in control, chow, and HFD-fed rats.

Serum aminotransferase increase is an index of hepatocyte injury. Consistent with our previous studies and others, we saw elevated serum ALT and ALP in animals with fatty livers (Toshikuni et al., 2013; Echeverria et al., 2018). Elevated ALT is also an indicator of insulin resistance. Fasting reduced serum ALT levels in HFD-fed rats. Such observations were reported in several studies showing improvement in liver enzymes (transaminase) after exercise and restricted diets (Vajro et al., 1994; Ueno et al., 1997). Except in HFD-fed rats, other experimental groups showed no decline in ALT levels with fasting, which is consistent with earlier reports (Krahenbuhl et al., 1991; Browning et al., 2012). Fasting for 48 h reduced ALP levels in all experimental animals, as has been reported in chow-fed control and CCl4-induced cirrhotic rats (Abraira et al., 1980; Krahenbuhl et al., 1991; Restellini et al., 2013; Fazeli et al., 2015).

Histological assessment of the H&E stained liver sections showed lipid accumulation after excessive alcohol intake or HFD consumption with the two models of fatty liver disease being essentially indistinguishable. This assessment was corroborated by biochemical quantitation which revealed a nearly 2.5-fold increase in hepatic TG in both models compared with pair-fed control or chow-fed rats.

Pathomechanisms that underlie the development of hepatic steatosis include increased *de novo* lipogenesis, increased circulating fatty acid uptake by the liver coupled with decreased fatty acid oxidation and reduced fat export as VLDL (Kharbanda et al., 2009; Wei et al., 2013; Osna et al., 2017; You and Arteel, 2019). For *de novo* lipogenesis, fatty acid synthesis is regulated by the enzyme fatty acid synthase (FAS). We observed increased expression of FAS mRNA in the livers of ethanol-fed rats compared with pair-fed controls, suggesting that increased *de novo* fatty acid synthesis contributes to ethanol-induced hepatic fat accumulation. Further, alcohol metabolism-induced increases in the NADH/NAD ratio could promote *de novo* fatty acid synthesis (Rasineni and Casey, 2012) and contribute to increased fat accumulation. Besides *de novo* fatty acid synthesis, a major physiological mechanism that contributes to fat accumulation in the liver is increased circulating NEFA uptake by the liver and its esterification with glycerol-3-phosphate to form TG (Alves-Bezerra and Cohen, 2017). Here, we observed increased serum NEFA in conjunction with an increased expression of hepatic fatty acid transporters FATP2 and CD36, indicating that increased hepatic uptake of circulating NEFA contributes to fat accumulation in ethanol-fed rats. Ethanol consumption also resulted in impaired triglyceride hydrolysis and fatty acid oxidation as indicated by a decline in the activation of the two major lipases (HSL and ATGL) as well as a decrease in PPARα, which regulates fatty acid oxidation. Collectively, it is the upregulation of lipogenic pathways, increased NEFA uptake, decreased hydrolysis and impaired fatty acid oxidation that all contribute to the ethanol-induced hepatic steatosis.

We observed that FAS mRNA expression is decreased in HFD-fed rats compared with chow-fed rats, indicating that *de novo* lipogenesis does not contribute significantly to HFD-induced hepatic steatosis. Several studies with dietary fat challenge in human subjects or experimental animals have similarly reported that the source of TG in hepatic tissue is mainly dietary lipids and that very little is from *de novo* lipogenesis (Donnelly et al., 2005; Delgado et al., 2009; Oosterveer et al., 2009; Duarte et al., 2014). Furthermore, HSL and ATGL activity were similar in both chow and HFD-fed animals and instead of an expected decrease, a moderate increase in PPARα expression was seen after HFD consumption. These results suggest that neither increased lipogenesis, impaired triglyceride hydrolysis nor decreased fatty acid oxidation contribute to the development of hepatic steatosis in HFD-fed rats. Rather, hepatic steatosis occurs through increased NEFA flow from the diet or adipose tissue and heightened uptake by the liver, facilitated by the elevated levels of fatty acid transporters CD36 and FABP4, as shown here. Other factors that may contribute to hepatic steatosis after HFD consumption are direct elongation and subsequent desaturation of dietary fatty acids in the liver (Oosterveer et al., 2009; Duarte et al., 2014).

Fasting for 48 h also revealed a differential effect on hepatic steatosis induced by ethanol or HFD consumption. While rats fed Lieber-DeCarli control, chow or high-fat diet showed accumulation of liver TG after 48 h of fasting, ethanol-fed rats showed a decrease compared with their fed counterparts. The fasting-induced change in hepatic lipids followed the same pattern as serum NEFA, which increased in all experimental groups compared to respective fed-groups except the 48 h fasted ethanol rats. Fasting reduced FAS expression while it increased PPARα expression in all experimental groups compared to respective fed groups. These findings corroborate previous studies, showing that fasting-induced glucocorticoid secretion promotes PPARα activation to utilize fat as energy source during fasting (Lemberger et al., 1994; Steineger et al., 1994). We also observed a decline in the activation of hepatic lipases, ATGL and HSL, in fasted control and HFD-fed rats whereas fasted chow-fed rats showed a decrease in only HSL activation. These results reveal that increases in lipogenic or decreases in fat oxidation pathways do not contribute to hepatic fat accumulation seen in rats fed the Lieber-DeCarli control, chow or high-fat diet after fasting. Rather, the increased liver delivery and uptake of the adipose-derived NEFA and its subsequent esterification combined with reduced breakdown of stored TG likely contributed to hepatic fat accumulation observed in these animals. While studies from other laboratories have indeed reported increases in hepatic triglyceride accumulation following 24 h or more of fasting in healthy humans and control rats (Moller et al., 2008; Browning et al., 2012; Geisler et al., 2016; Pantaleao et al., 2017), ours is the first report to show that fasting for 48 h increases hepatic steatosis in an HFD-associated NAFLD (non-alcoholic fatty liver disease) animal model.

The decrease in hepatic steatosis in alcohol-fed rats after 48 h fasting compared to fed-counterparts could be due to the combined effects of (i) abstinence-related recovery of the liver and adipose tissue, (ii) an increase in liver fatty acid oxidation as evident by increased PPARα, and (iii) the absence of further increases in the supply of adipose-derived NEFA to the liver because of an inadequate starvation-induced lipolytic stimulus as observed in pair-fed control rats.

It is noteworthy that there was no further change in the hepatic p-ATGL (active form) between fed and fasted ethanol rats, and that p-HSL (active form) decreased in fasted ethanol-fed rats compared with fed-counterparts. This decrease in active HSL after fasting likely contributes to the residual fat accumulation due to deceleration of lipolysis in the livers of fasted ethanol-fed rats.

In congruence with previous reports, ethanol-fed rats showed decreased epididymal white adipose tissue (eWAT) weight and adipocyte size while both were significantly elevated in HFD-fed rats compared with their respective controls. In correlation with adipocyte size, we observed increased HSL enzyme activity and decreased expression of PPARγ, which induces adipose differentiation and increases its fat storing capacity in the eWAT of ethanol-fed rats compared with control. These ethanol-induced changes in eWAT promote adipose lipolysis, causing a rise in adipose-derived serum NEFA levels as reported in our recent study (Rasineni et al., 2019a). Fasting pair-fed control rats reduced eWAT PPARγ expression while it increased adipose p-HSL levels, thereby enhancing lipolysis, as evident by reduced adipocyte size, and subsequently, an elevation in serum NEFA levels. These results were as expected. However, this normal fasting-induced lipolytic response was absent in ethanol-fed rats, with no change in PPARγ, active HSL or serum NEFA levels between the fed and fasted states.

We observed that the dietary fat mainly contributed to the higher serum NEFA levels in HFD-fed rats compared with chow-fed rats. However, the increased HSL activation seen in eWAT of HFD-fed rats may have also contributed to the rise in NEFA. Fasting for 48 h induced similar responses in the eWAT of chow and HFD-fed rats and caused a further increase in HSL activation to promote eWAT lipolysis (as judged by the decrease in adipocyte size compared to fed-counterparts) ultimately leading to elevated adipose-derived serum NEFA levels.

Increased fat accumulation makes the liver susceptible to inflammatory mediators, thereby, promoting the development of advanced liver injury. We observed elevated levels of the chemokine MCP-1 in livers of ethanol- and HFD-fed rat which is consistent with previous reports (Ito et al., 2007; Mandrekar et al., 2011; Gao and Tsukamoto, 2016). However, we observed no histological evidence of inflammation. This may be because MCP-1 not only plays a role in macrophage recruitment and inflammation but also directly plays a significant role in the development of hepatic steatosis via affecting fatty acid metabolism (Mandrekar et al., 2011). FFAs regulate MCP-1 expression (Yeop Han et al., 2010; Asterholm et al., 2012), which we believe may be contributing to the increase in this chemokine level in livers of HFD- and ethanol-fed rats. Upon fasting, all other experimental rats, except for the ethanol-fed animals, showed increased hepatic MCP-1 levels, which is in line with the increased flow of FFAs seen in these other experimental groups after 48 h fasting.

In summary, although AFL and NAFL feeding models showed similar histology, the physiological mechanisms in fatty liver development in each are different. Increased hepatic *de novo* fatty acid synthesis, higher uptake of adipose-derived NEFA and impaired fatty acid breakdown contribute to the development of AFL. In the development of NAFL, however, increased dietary fatty acid uptake by the liver plays a major role. Likewise, the response of each model to fasting is also diverse. While hepatic steatosis decreased after fasting in ethanol-fed rats, animals with NAFL had increased hepatic steatosis compared with their fed-counterparts. This rise in hepatic steatosis after 48 h fasting of HFD-fed rats was likely due to increased delivery to the liver and enhanced hepatic uptake and esterification of adipose-derived NEFA combined with reduced TG lipolysis. The decrease in hepatic steatosis in AFL rats after 48 h fasting likely resulted from a combined effect of abstinence-related recovery, an increase in fatty acid oxidation and the absence of any further increase in the supply of adipose-derived NEFA to the liver.

## Data Availability Statement

The original contributions presented in the study are included in the article/supplementary material, further inquiries can be directed to the corresponding author.

## Ethics Statement

The animal study was reviewed and approved by the Institutional Animal Care and Use Committee at the VA NWIHCS Research Service.

## Author Contributions

KR: funding acquisition, conception, design of the study, data analysis and interpretation, and writing the original draft. CJ: performing the experiments, data acquisition, and data analysis. PT, JK, ES, and SS: aided in performing the experiments. GT: histological evaluation. TD: helped with interpretation of the data and editing the manuscript. MM: funding acquisition. KK: funding acquisition, data interpretation, and review and editing the manuscript. CC: funding acquisition, supervision of the project, and review and editing the manuscript. All authors contributed to the article and approved the submitted version.

## Conflict of Interest

The authors declare that the research was conducted in the absence of any commercial or financial relationships that could be construed as a potential conflict of interest.
